# The prognostic significance of *β*-Catenin expression in patients with nasopharyngeal carcinoma: A systematic review and meta-analysis

**DOI:** 10.3389/fgene.2022.953739

**Published:** 2022-08-09

**Authors:** Liu-Qing Zhou, Jin-Xiong Shen, Tao Zhou, Chun-Li Li, Yao Hu, Hong-Jun Xiao

**Affiliations:** ^1^ Department of Otorhinolaryngology, Union Hospital, Tongji Medical College, Huazhong University of Science and Technology, Wuhan, China; ^2^ Department of Otorhinolaryngology, Wuhan First Hospital/Wuhan Hospital of Traditional Chinese and Western Medicine, Wuhan, China; ^3^ Department of Otorhinolaryngology, The Central Hospital of Wuhan, Wuhan, China

**Keywords:** *β*-catenin, nasopharyngeal carcinoma, prognosis, meta-analysis, os

## Abstract

**Background:** *β*-Catenin has been recently identified as a promising novel therapeutic target and prognostic marker in different types of cancer. Here, we conduct a meta-analysis to better clarify the correlation between *β*-Catenin expression and survival outcomes in nasopharyngeal carcinoma (NPC) patients.

**Patients/methods:** Following the Preferred Reporting Items or Systematic Reviews Meta Analyses (PRISMA) 2020 guidelines, the PubMed, Embase, Web of Science, Cochrane Library, Chinese National Knowledge Infrastructure (CNKI) and Wanfang databases were systematically searched for relevant studies to explore the prognostic significance of *β*-Catenin in NPC. Pooled hazards ratios (HRs) and 95% confidence intervals (CIs) were used to estimate the association of *β*-Catenin expression with survival outcomes in NPC patients. Odd ratios (ORs) and 95% CIs for clinicopathological characteristics were also statistically analyzed.

**Results:** Eight studies involving 1,179 patients with NPC were ultimately included in the meta-analysis. Pooled analysis indicated that elevated *β*-Catenin expression was significantly associated with poor OS (HR = 2.45, 95% CIs: 1.45–4.16, *p* = 0.001) and poor DFS/PFS (HR 1.79, 95% CIs: 1.29–2.49, *p* = 0.000). Furthermore, *β*-cadherin was signifcantly associated with higher TMN stages (OR = 5.10, 95% CIs 2.93–8.86, *p* = 0.000), clinical stages (OR = 5.10, 95% CIs 2.93–8.86, *p* = 0.000) and lymph node metastasis (LNM) (OR = 5.01, 95% CIs 2.40–10.44, *p* = 0.000).

**Conclusions:** This study demonstrated that for NPC, patients with elevated *β*-Catenin expression are more likely to have poor survival.

## Introduction

Nasopharyngeal carcinoma (NPC) is one of the most common types of head and neck tumors and shows remarkable differences in geographic and racial distribution (Stransky et al., 2011). NPC is prevalent in Southeast Asia, especially in Southern China, the Arctic region and North Africa (Chang and Adami, 2006). Risk factors for NPC include male sex, EBV infection, Cantonese ethnicity, salt-preserved fish consumption, low fresh fruit and vegetable intake, and smoking, among others. Irrespective of the progress in radiation therapy and potent chemotherapy, approximately 5%–15% local recurrence and 15%–30% distant metastasis rates remain the main causes of failure after NPC treatment (Lee et al., 2015). Clinical staging is essential for the prognosis of NPC; however, patients at the same clinical stage may have different prognoses. In general, the current staging system is inadequate to predict survival due to variations in treatment outcomes. Hence, it is necessary to identify more reliable prognostic factors to improve the prognosis of NPC.

β-Catenin was first characterized as a family of cell-cell adhesion molecules dependent on Ca^2+^ that are present in most cell types, and it was also shown to have more detailed specificity with regard to cell-cell aggregation patterns and segregation during development (Takeichi, 1990). *β*-Catenin is one of the hallmarks of the epithelial-mesenchymal transition, which is important for early tumor metastasis and invasion (Thiery and Sleeman, 2006). It also plays a crucial role in the Wnt/β-Catenin signaling pathway, which is one of the most important signaling pathways involved in many human malignancies, and might participate in the development of various cancers and tumors (Anastas and Moon, 2013). Indeed, aberrant activation of Wnt/β-Catenin signaling is found in various types of human cancer, including osteosarcoma, lung cancer, colorectal cancer, renal cell carcinoma, breast cancer, and hepatocellular cancer, among others (Kim et al., 2002; Hoang et al., 2004; Arai et al., 2014; Jang et al., 2015; Fu et al., 2016).

Numerous studies have focused on the identification of new prognostic markers that can be used for cancer monitoring and detection. An association between *β*-Catenin expression and survival has been shown in NPC (Wang et al., 2009; Luo et al., 2012; Xu et al., 2013; Pang et al., 2019). Although many studies have reported an association between *β*-Catenin expression and NPC patient survival, the results are still controversial and ambiguous. For example, Jin et al. (2019), Sun et al. (2017), Wang et al. (2017) found that *β*-Catenin is highly expressed in NPC and is a potential risk factor that leads to an unfavorable survival prognosis in these patients. However, contradictory results were reported by Hao et al. (2014), Galera-Ruiz et al. (2011), who found no association between *β*-Catenin and survival in NPC patients compared with normal controls. In this study, we conducted a meta-analysis based on PubMed, Embase, Web of Science, Cochrane Library, Chinese National Knowledge Infrastructure (CNKI) and Wanfang databases to statistically assess the association between *β*-Catenin and the prognosis of NPC patients.

## Methods

### Search strategy

Following the Preferred Reporting Items or Systematic Reviews Meta Analyses (PRISMA) 2020 guidelines, electronic searches for relevant studies were performed in the PubMed, Web of Science, EMBASE, Cochrane Library, Chinese National Knowledge Infrastructure (CNKI) and Wanfang database until 1 March 2022 (Page et al., 2021). The search terms of PubMed were “((((((((((((((((((((Nasopharyngeal Neoplasm) OR (Neoplasm, Nasopharyngeal)) OR (Neoplasms, Nasopharyngeal)) OR (Nasopharynx Neoplasms)) OR (Nasopharynx Neoplasm)) OR (Neoplasm, Nasopharynx)) OR (Neoplasms, Nasopharynx)) OR (Cancer of Nasopharynx)) OR (Nasopharynx Cancers)) OR (Nasopharyngeal Cancer)) OR (Cancer, Nasopharyngeal)) OR (Cancers, Nasopharyngeal)) OR (Nasopharyngeal Cancers)) OR (Nasopharynx Cancer)) OR (Cancer, Nasopharynx)) OR (Cancers, Nasopharynx)))) OR (Cancer of the Nasopharynx)) AND ((((((prognosis) OR (outcome)) OR (recurrence)) OR (survival)) OR (mortality)) OR (progression))) AND ((Catenin, beta) OR (beta-Catenin)) ” The EMTREE terms were as follows “(‘nasopharynx cancer'/exp OR rhinopharyngioma OR ‘cancer, nasopharynx’ OR ‘epipharynx cancer’ OR ‘nasopharyngeal cancer’ OR ‘rhinopharynx cancer’) AND (prognosis OR outcome OR recurrence OR survival OR mortality OR progression) AND (‘beta catenin’/exp OR “catenin beta”).” Furthermore, the reference lists of retrieved articles were manually searched for additional articles. If several publications reported the same patient populations, the most complete study was enrolled to avoid duplication.

### Selection criteria

This meta-analysis was limited to publications about the association between NPC and *β*-Catenin. The inclusion criteria of the meta-analysis were as follows: 1) all patients diagnosed with NPC; 2) *β*-Catenin was evaluated in both samples of NPC and normal controls; 3) the study revealed the association between *β*-Catenin and survival of NPC; 4) sufficient statistical analysis, including hazard ratios (HRs), odds ratios (ORs) and their 95% confidence intervals (95% CIs) were reported. The exclusion criteria were as follows: 1) studies without sufficient data for meta-analysis; 2) abstracts, reviews, letters, expert opinions; 3) studies about cell lines, *in vivo*/vitro studies, and human xenografts. If several studies reported the same cohort, we used the most recent one in our meta-analysis.

### Data extraction

First, we inspected duplicates and removed repeated papers. Then, we carefully perused the titles and abstracts of the papers. Finally, full articles were selected to include appropriate studies. Two researchers independently evaluated the literature using the inclusion and exclusion criteria (LQ Zhou and Y Hu). Any discrepancy in assessment was resolved by consulting with a third researcher (HJ Xiao). The authors of the studies were contacted by e-mail to request data or additional information for meta-analysis calculations. Eligible studies were reviewed by two reviewers (LQ Zhou and Y Hu) independently. The Newcastle–Ottawa Scale (NOS) (Peterson et al., 2011) was included to assess the quality of the included publications, and a star system (maximum is nine stars) was adopted to evaluate a study in three domains: comparability of study groups, selection of participants and ascertainment of outcomes of interest. Scores of NOS ≥6 indicated high-quality studies. Reporting recommendations for tumor marker prognostic studies (REMARK) were also applied to evaluate study quality in cancer-related meta-analyses (Sauerbrei et al., 2018).

The following information was extracted from each publication: 1) first author’s name, year, cancer type, country of the population, patient age, sample size, publication journal; 2) survival data including overall survival (OS), disease-free survival (DFS) and progression-free survival (PFS) (OS was detected from the date of medical treatment to the date of the last follow-up or death of patient; PFS was detected from the date of treatment to the date of death or recurrence tumor from any cause; DFS was detected from the date of diagnosis to the date of relapse, progression, death, or last follow-up visit and similarly censored at last follow-up visit); 3) The number of patients in each group was divided according to the TMN stages, clinical stages, the presence or absence of lymph node metastasis (LNM), gender and the number of patients with high or low *β*-catenin expression in each group. 4) Methods and cut-off value (Table 1).

**TABLE 1:** Characteristics of the studies examined in the meta-analysis. NR, not reported; IHC, Immunohistochemistry; RT-qPCR, Reverse transcription-quantitative polymerase chain reaction.

Author	Year	Country	Sample size	Age	Follow-up (month)	Survival analysis	Methods	Cut-off value	NOS/REMARK score
Hao	2014	Canada	279	51.7 (18-85)	48 (3-120)	OS, DFS	IHC	NR	6/15
Jin	2019	China	164	45.3 (24-70)	49.2 (9-60)	OS, DFS, DMFS, LRFS	IHC, RT-qPCR	50%	7/13
Pang	2019	China	175	NR (22-69)	NR (36-48)	OS	IHC	75%	7/14
Sun	2017	China	128	NR	NR	OS, PFS	IHC, RT-qPCR	50%	7/12
Wang	2009	China	111	47 (18-71)	65 (8-88)	PFS	IHC	50%	7/11
Wang	2017	China	163	NR	NR	OS	IHC	70%	8/11
Xu	2013	China	148	NR	78 (10-125)	OS	IHC	NR	7/12
Luo	2012	China	122	47.2 (15-73)	51.9 (8-92)	OS	IHC	50%	7/12

### Statistical analysis

Pooled HRs, ORs and their 95% CIs were directly obtained or estimated by *p* values and other published data following Parmer’s methods from the primary studies (Ambrosio et al., 2014). Statistical heterogeneity among the included studies was assessed by the χ^2^-based Q test and I^2^ test (Higgins et al., 2003). The fixed-effect model was used for analysis in the absence of significant heterogeneity between studies (*p* > 0.10, I^2^ = 0%); we adopted the random-effects model if significant heterogeneity was present. We also performed sensitivity analysis to investigate the influence of each individual study on the overall pooled results. Begg’s test and Egger’s test were applied to detect publication bias (*p* > 0.05 indicated no publication bias). All statistical analyses were performed using STATA statistical software version 12.0 (STATA, College Station, TX).

## Results

### Study selection and characteristics

**FIGURE 1:**
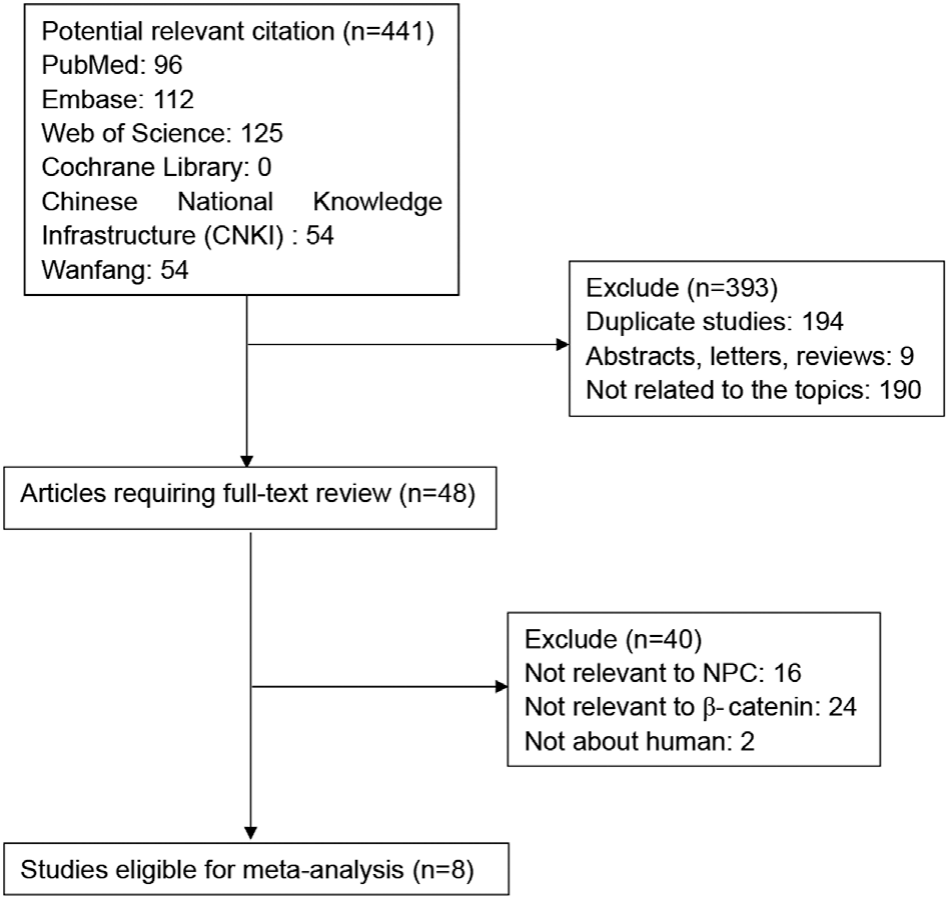
Flow diagram of the selection of relevant studies for the meta-analysis.

As shown in Figure 1, a total of 312 potential publications were initially identified by searching the PubMed, Web of Science, EMBASE, Cochrane Library, Chinese National Knowledge Infrastructure (CNKI) and Wanfang databases. Following exclusion of duplicates (*n* = 194), abstracts, letters and reviews (*n* = 9), and studies not related to the topics (*n* = 190), the remaining potentially relevant studies (*n* = 48) were further identified by reading their full texts. 40 studies did not provide specific data regarding NPC or *β*-Catenin and therefore were excluded. Finally, eight studies between 2009 and 2020 with a total of 1179 NPC patients were included in our meta-analysis.

The study characteristics are summarized in Table 1. All of the eight publications involved >100 patients. Seven studies including 1,068 patients reported OS, 2 studies including 443 patients reported DFS, and 2 studies including 239 patients reported PFS. All HRs, ORs and 95%CIs values were directly reported in the original study. NOS scores for all publications were above 6, and REMARK scores were between 11-15.

### Association between *β*-Catenin and survival in nasopharyngeal carcinoma patients

**FIGURE 2:**
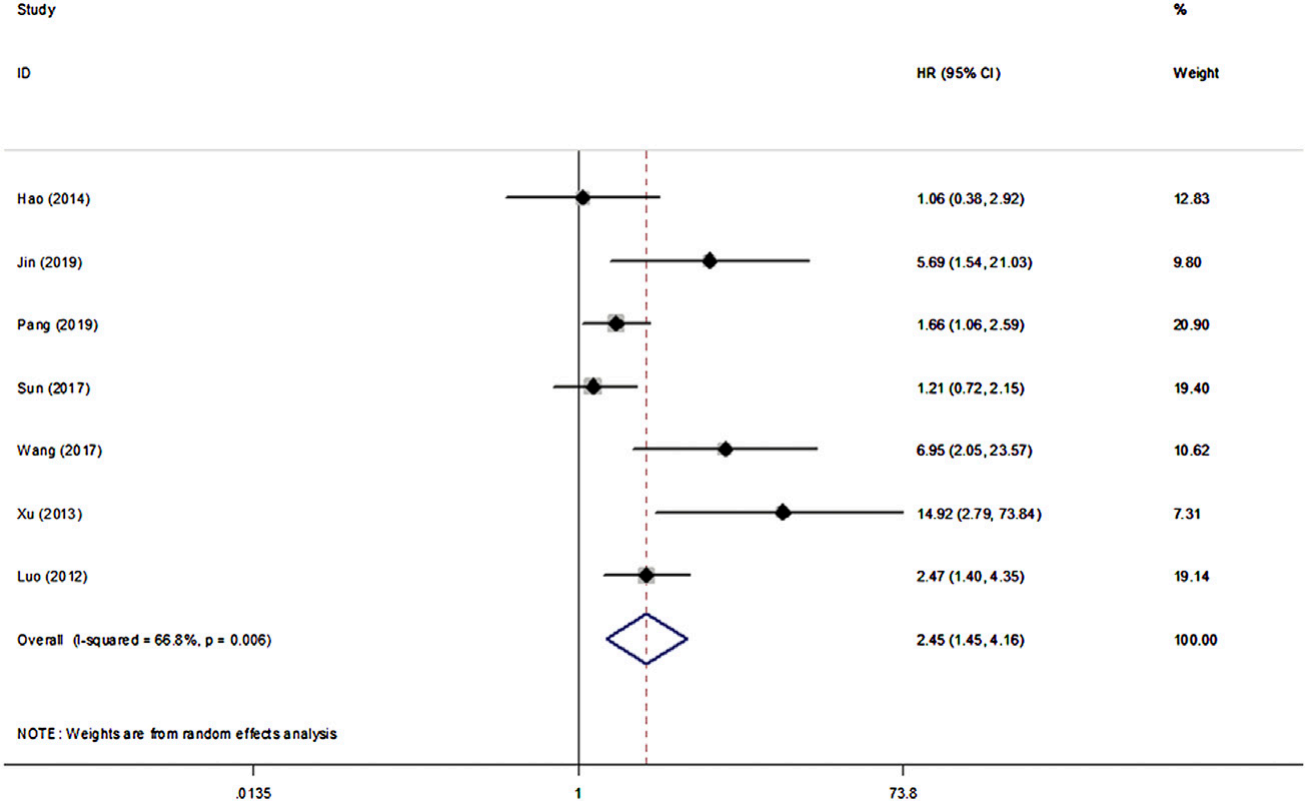
Forest plot indicating the association between *β*-Catenin expression and OS in NPC.

**FIGURE 3:**
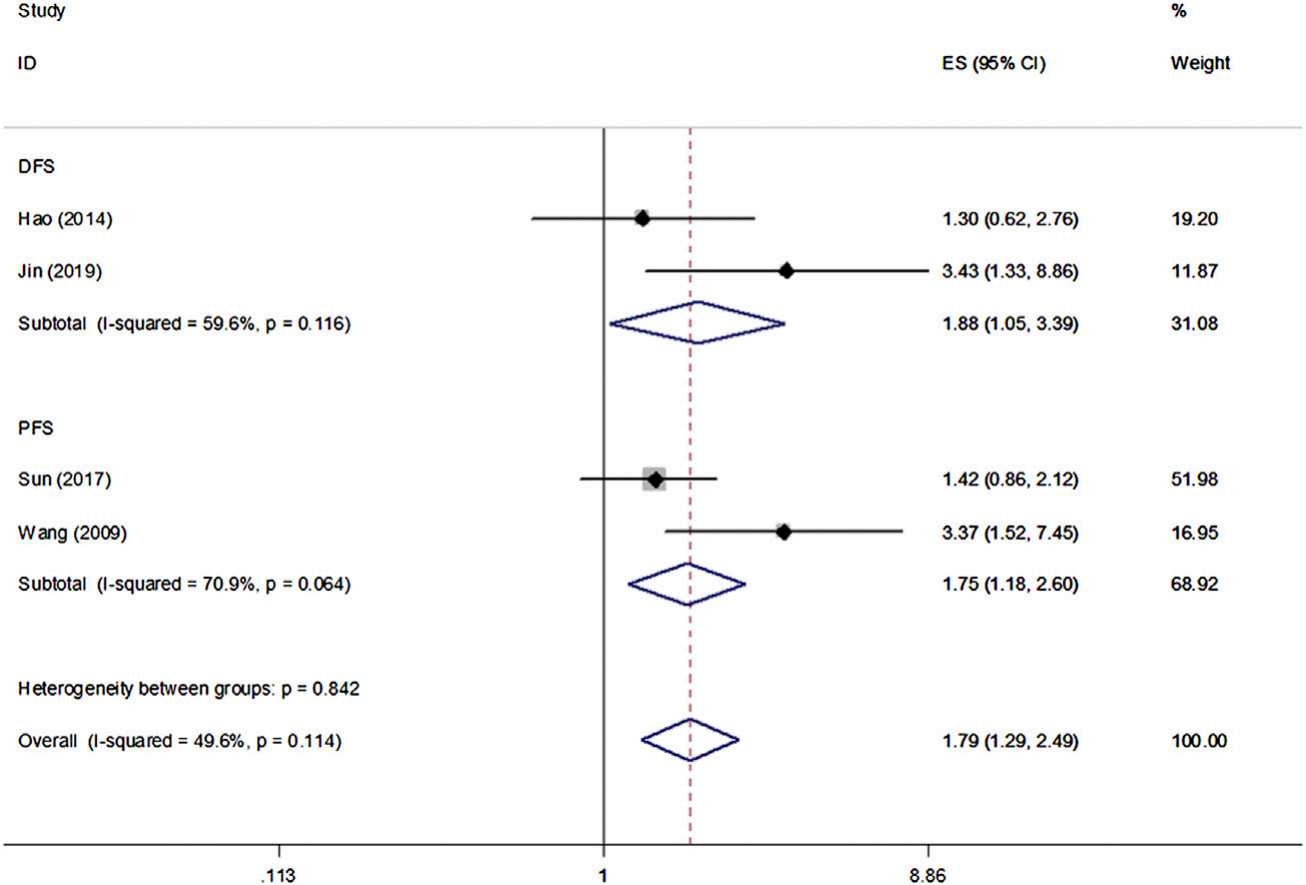
Forest plot examining the association between *β*-Catenin expression and DFS/PFS in NPC.

All eight studies in our analysis reported the association between *β*-Catenin and the OS, DFS and PFS of patients with NPC. Heterogeneity among the publications was significant based on the Q test (*p* < 0.1). Hence, the random-effect model was adopted and showed that *β*-Catenin was significantly associated with shorter OS in NPC (HR = 2.45, 95% CIs: 1.45–4.16, *p* = 0.001). Medium heterogeneity was noted between *β*-Catenin expression and OS (I^2^ = 66.8%, P_heterogeneity_ = 0.006) (Figure 2). Furthermore, two studies including 433 patients reported DFS, and two studies including 239 patients reported PFS. A significant correlation between *β*-Catenin and shorter DFS/PFS (HR = 1.79, 95% CIs: 1.29–2.49, *p* = 0.000) was observed, with low heterogeneity (I^2^ = 49.6%, P_heterogeneity_ = 0.114) (Figure 3).

### Association between *β*-cadherin and nasopharyngeal carcinoma patients outcomes

**FIGURE 4:**
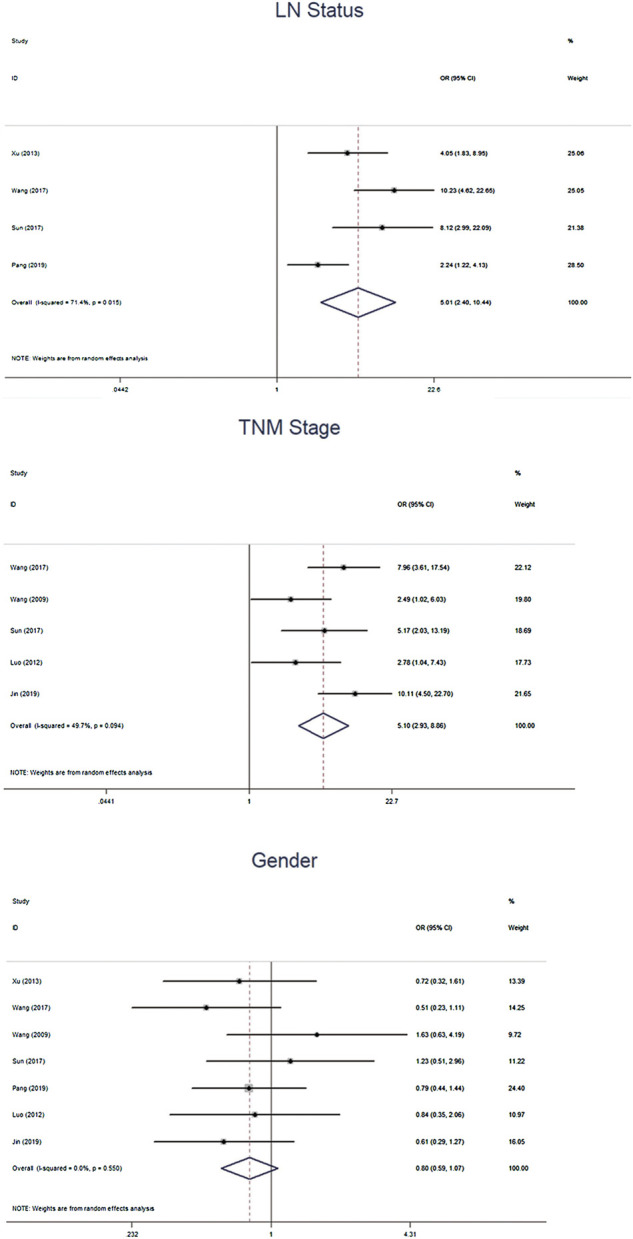
Forest plot examining the association between *β*-cadherin and NPC patients outcomes.

We further calculated the pooled ORs and the 95% CIs to evaluate the association between *β*-catenin and NPC outcomes: gender (female vs male), TMN stage (T3–4 vs T1–2), clinical stage (T3–4 vs T1–2) and lymph node (LN) status (LNM vs No LNM). The pooled analysis showed that *β*-cadherin was signifcantly associated with higher TMN stages (OR = 5.10, 95% CIs 2.93–8.86, *p* = 0.000) and LNM (OR = 5.01, 95% CIs 2.40–10.44, *p* = 0.000). However, *β*-cadherin was not signifcantly correlated with gender (OR = 0.80, 95% CIs 0.59–1.07, *p* = 0.135) (Figure 4).

### Sensitivity analysis

**FIGURE 5:**
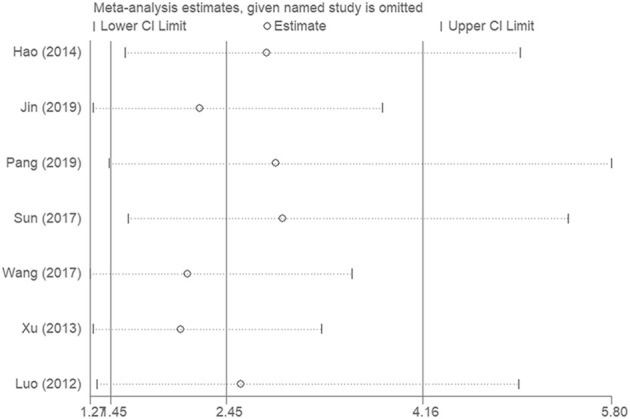
Sensitivity analyses were conducted to evaluate the impact of each single study on the overall effect.

Sensitivity analysis was conducted to evaluate the impact of each single study on the overall effect. As depicted in Figure 5, the analysis did not detect a single study that significantly altered the combined results. Overall, the pooled effect size of our meta-analytic results was stable and reliable.

### Publication bias

**FIGURE 6:**
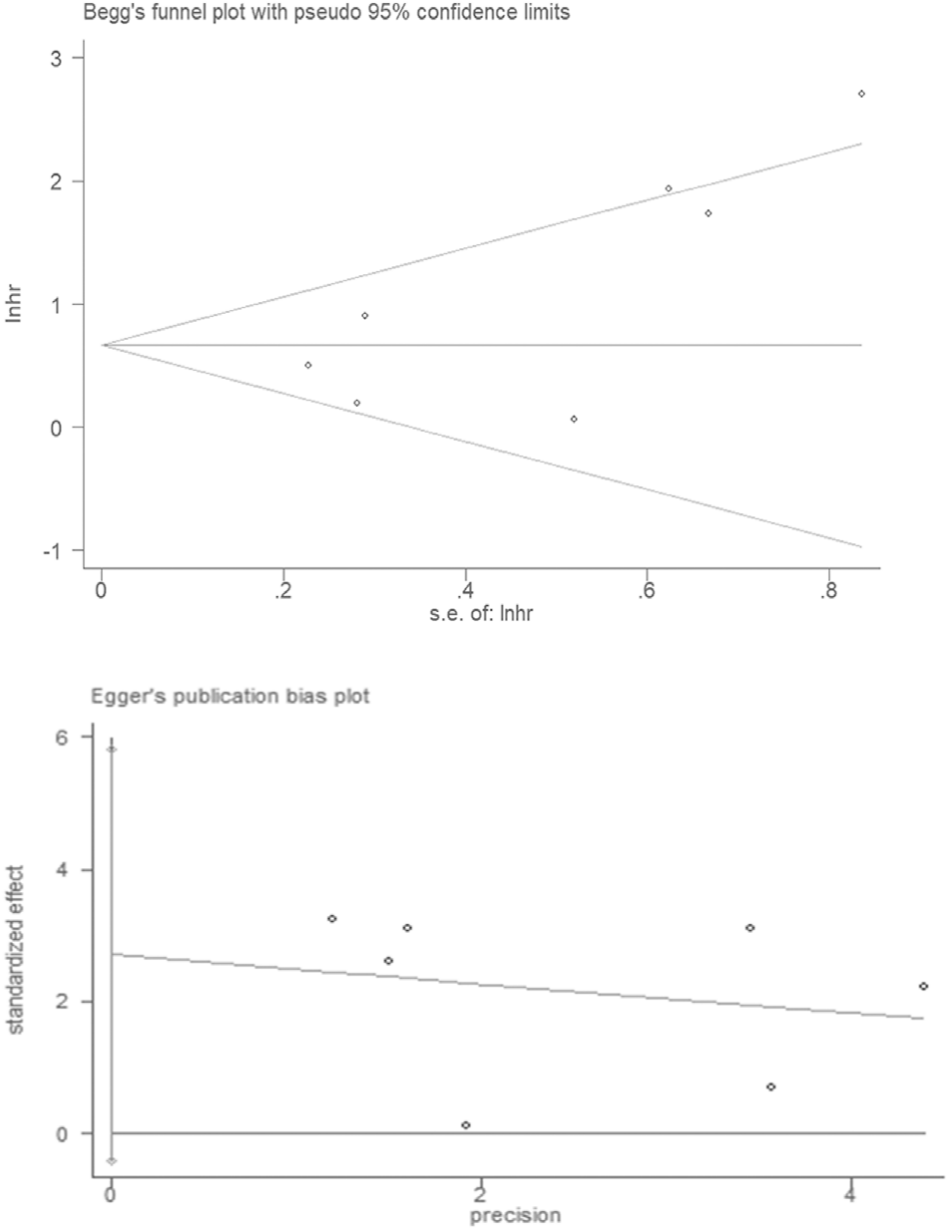
Publication bias in the enrolled studies. Publication bias was assessed using Begg’s funnel plots and Egger’s test.

Publication bias was assessed by using Begg’s funnel plots and Egger’s test. The results were quite symmetric, with those based on Begg’s funnel plot (*p* = 0.077) and Egger’s test (*p* = 0.077) revealing no publication bias among the studies (Figure 6).

### Meta-regression analysis

Medium heterogeneity was noted between *β*-Catenin expression and OS (I2 = 66.8%, Pheterogeneity = 0.006). Hence, the meta regression analyses were used to explain statistical heterogeneity. The HR was not modified by the year of publication, female ratio (%), area, sample size, or quality score, this result does not fully explain the medium level of heterogeneity observed.

## Discussion

The present study is the first meta-analysis including eight published studies with 1,179 patients to provide useful information for clinical decision-making in NPC. *β*-Catenin was significantly associated with shorter OS in NPC patients, with HR values of 2.45. Significant correlation between *β*-Catenin and shorter DFS/PFS (HR 1.79) was also observed. Furthermore, our results also demostrated that *β*-cadherin was signifcantly associated with higher TMN stages, clinical stages and LNM. These results confirm the clinical value of *β*-Catenin in NPC. NPC tumor cells invade adjacent tissues or metastasize to regional lymph nodes at an early stage of NPC development (Wei and Mok, 2007), though the exact mechanism underlying the process remains unknown. It has been reported that cell–cell adhesion molecules, cytokines and the matrix metalloproteinase family may be involved in adjacent invasion and distant metastasis. *β*-Catenin is a key mediator in the cadherin-Catenin complex, which is essential for connecting the actin filaments of cells to the cell-cell interface at adherent junctions (Anastas and Moon, 2013); it is also a key mediator of canonical signaling in the Wnt/β-Catenin pathway. *β*-Catenin can accumulate in both the cytoplasm and nucleus (Khramtsov et al., 2010), and it helps to promote the progression of tumors by suppressing T-cell responses (Hong et al., 2015). Gene mutations or aberrant activation of Wnt receptors activate Wnt/β-Catenin signaling and trigger tumorigenesis in the skin, colon, liver, bone marrow, breast, and possibly other tissues (Fodde and Brabletz, 2007; Monga, 2015). In addition, *β*-Catenin plays roles in maintaining the stemness of normal intestinal cells, and high-level nuclear localization and cytoplasmic expression promote cancer cell proliferation and survival (Valkenburg et al., 2011). *β*-Catenin is high expressed when Wnt/β-catenin signal is aberrantly activated, it activates numerous Wnt pathway downstream proliferation signals, including c-Myc and cyclin D1 and finally accelerates cell cycle, facilitates cell proliferation and migration, which induced to poor diagnosis of NPC (Alamoud and Kukuruzinska, 2018).

Targeted therapies have produced striking benefits for cancer patients. The Wnt/β-Catenin pathway has been proven to play a key role in various kinds of carcinomas (Sanchez-Vega et al., 2018). Therefore, this signaling pathway is a preferable target for fighting cancer. Although there are no drugs specifically inhibiting this signaling pathway approved for the clinic, intensive efforts have been made in signaling pathway development. Wnt/β-Catenin pathway inhibitors can be classified into five categories according to their properties: peptides, small molecules, antibodies, natural compounds and RNA interference (Cui et al., 2018). There are already some ongoing phase 1/2 trials with Wnt/β-Catenin pathway inhibitors in metastatic colorecta, head and neck cancers, breast cancers and some other cancers (Krishnamurthy and Kurzrock, 2018). These trials provide proof that in certain patients, cancer can be treated with Wnt/β-Catenin inhibitors. According to our results, Wnt/β-Catenin inhibitors may constitute therapeutics against NPC.

Nevertheless, the present meta-analysis contains several limitations. First, significant heterogeneity was noted in the association between *β*-Catenin and the OS of patients with NPC. The heterogeneity of the population was most likely due to differences in the baseline characteristics of the included patients (age, race, and tumor stage), the duration of follow-up, the method of mutation detection, and other parameters. A random-effects model was employed to minimize the effects of these differences. Second, the number of articles used for assessing the association between *β*-Catenin and the prognosis of NPC patients was limited in the present meta-analysis. Therefore, additional studies are required to produce accurate conclusions. Finally, our results may overestimate the prognostic significance of *β*-Catenin to some extent because the majority of the included studies reported positive results.

In summary, we searched electronic databases, and a total of 1,179 patients in eight studies were enrolled for meta-analysis, demonstrating that patients with elevated *β*-Catenin expression are more likely to have poorer prognosis. Taken together, our meta-analysis results suggest that *β*-Catenin has prognostic value for NPC. However, studies with larger sample sizes are needed to obtain more representative and precise results.

## Supplementary material

Supplementary Table S1 Quality assessment based on the Recommendations for Tumor Marker Prognostic Studies (REMARK).

Supplementary Table S2 PRISMA_2020_checklist.

Supplementary Table S3 PRISMA_2015_checklist.

Supplementary Table S4 Quality assessment based on the Newcastle-Ottawa Scale (NOS).
