# Supplementary material for: The prognostic significance of β-Catenin expression in patients with nasopharyngeal carcinoma: A systematic review and meta-analysis
Source: Front Genet. 2022 Aug 9;13:953739. doi: 10.3389/fgene.2022.953739 (PMC9400172; doi:10.3389/fgene.2022.953739)
Supplement: Supplementary file 4 [file Table4.DOCX]

| **Author** | **Year** | **Selection** | **Comparability** | **Outcome** | **NOS score** |
| --- | --- | --- | --- | --- | --- |
| Hao [23] | 2014 | 3 | 2 | 1 | 6 |
| Jin [20] | 2019 | 3 | 2 | 2 | 7 |
| Pang [17] | 2019 | 3 | 2 | 2 | 7 |
| Sun [21] | 2017 | 3 | 3 | 1 | 7 |
| Wang [18] | 2009 | 3 | 2 | 2 | 7 |
| Wang [22] | 2017 | 3 | 3 | 2 | 8 |
| Xu [19] | 2013 | 3 | 2 | 2 | 7 |
| Luo [16] | 2012 | 3 | 3 | 1 | 7 |
